# VX-984 is a selective inhibitor of non-homologous end joining, with possible preferential activity in transformed cells

**DOI:** 10.18632/oncotarget.25383

**Published:** 2018-05-25

**Authors:** Atif J. Khan, Sarah M. Misenko, Aditya Thandoni, Devora Schiff, Sachin R. Jhawar, Samuel F. Bunting, Bruce G. Haffty

**Affiliations:** ^1^ Department of Radiation Oncology, Memorial Sloan Kettering Cancer Center, New York, NY 10011, USA; ^2^ Department of Radiation Oncology, Rutgers-Robert Wood Johnson Medical School, Piscataway, NJ 08854, USA; ^3^ Department of Molecular Biology and Biochemistry, Rutgers, The State University of New Jersey, Piscataway, NJ 08854, USA

**Keywords:** DNA-PK, non-homologous end joining, DNA repair, double-strand break repair, radiation therapy

## Abstract

**Purpose:**

DNA double-strand breaks (DSBs) can be repaired by non-homologous end joining (NHEJ) or homologous recombination (HR). We demonstrate the selectivity of VX-984, a DNA-PK inhibitor, using assays not previously reported.

**Experimental Design:**

The class switch recombination assay (CSR) in primary B cells was used to measure efficiency of NHEJ. A cellular reporter assay (U2OS EJ-DR) was used to assess the efficiency of HR and NHEJ in cells treated with VX-984. Immunofluorescence assays (IF) evaluated γ-H2AX foci for DSB repair kinetics in human astrocytes and T98G glioma cells. Western blotting was used to evaluate phosphorylation of DNA-PKcs substrates.

**Results:**

We found a dose-dependent reduction in CSR efficiency with VX-984, and through the EJ-DR assay, dramatic dose-dependent increases in HR and mNHEJ. Immunofluorescence assays showed an inability of malignant cells to resolve γ-H2AX foci in the presence of VX-984. Radiation-induced phosphorylation of DNA-PK substrates was further reduced by treatment with VX-984.

**Conclusions:**

VX-984 efficiently inhibits NHEJ, resulting in compensatory increases in alternative repair pathways, increases DSBs, and appears to affect transformed cells preferentially.

## INTRODUCTION

The maintenance of chromosomal structure and proper replication of genetic information is critical in ensuring the survival of higher order eukaryotic species [1, 2]. Cells have evolved complex pathways to reduce the impact of innumerable DNA lesions and mutations caused by various DNA damaging agents in the environment [1–3]. The most lethal and toxic of lesions to DNA are double-strand breaks (DSBs) [2–4]. DSBs can be caused through exposure to ionizing radiation, endogenous metabolic processes, or numerous DNA-damaging reagents [5–7]. The processes that enable detection and resolution of these DSBs are collectively referred to as the DNA-damage response (DDR), and have been studied extensively [1, 2, 8]. Broadly speaking, the two primary DSB repair pathways used by eukaryotic cells are the non-homologous end joining (NHEJ) and homologous recombination (HR) pathways [1, 6–9]. HR employs a sister chromatid or sequence from a homologous chromosome to serve as a template for repair, thus creating a less error-prone system. However, HR is mostly restricted to the S/G2 phases of the cell cycle when a sister chromatid is available to be used as a template [1, 3, 7]. NHEJ, which can take place throughout the cell cycle, is more error-prone but more frequently invoked, even among cells that are eligible for HR. DSB repair pathway selection in cells that have the capacity to use both HR and NHEJ is not well understood.

A key factor in the NHEJ process is DNA-PK, a complex with serine/threonine kinase activity that is comprised of the heterodimeric Ku70/80 proteins and the catalytic subunit DNA-PKcs [4, 5, 8, 10, 11]. As a member of the phosphatidylinositol 3-kinase related family of protein kinases (PI3KK), DNA-PK, along with related proteins such as ATR and ATM, initiates the signal cascade of responses required for DNA repair [4, 11, 12]. Through a series of interactions and autophosphorylation reactions at the ABCDE (Thr2609) and PQR (Ser2056) clusters, DNA-PK phosphorylates other proteins committed to the NHEJ cascade of events [4–6, 8, 10]. The activation of DNA-PK is an early and critical event in the progress of NHEJ, and inhibition of this enzyme as a therapeutic strategy has been of interest [13, 14]. In particular, combining inhibitors of DDR with conventional therapies that cause DNA DSBs, such as radiation therapy (RT), could lead to previously unseen efficacy in the killing of malignant cells. The dominant issue that leads to symptoms and death in certain malignancies, such as high-grade astrocytoma/glioblastoma, is local progression (despite surgery/RT/chemotherapy). Thus, improving the efficacy of RT could improve local control and lead to dramatic improvements in patient survival.

VX-984 is a novel drug candidate that is intended to act as a selective inhibitor of DNA-PKcs. Radiation therapy with VX-984 has been shown to enhance cell death *in vitro* and *in vivo* assays [15]. In this report, we further demonstrate the selectivity of VX-984 in its inhibition of NHEJ using assays that have not been previously reported. Although RT is a localized treatment that can be spatially restricted to malignant areas, enhanced treatment-related toxicity is a potential concern when combining RT with an agent that inhibits DDR. For this reason, we evaluated relative DSB repair dynamics in untransformed and transformed astrocytes treated with VX-984.

## RESULTS

### VX-984 is a potent inhibitor of NHEJ as measured with the class switch recombination assay

**Figure 1:**
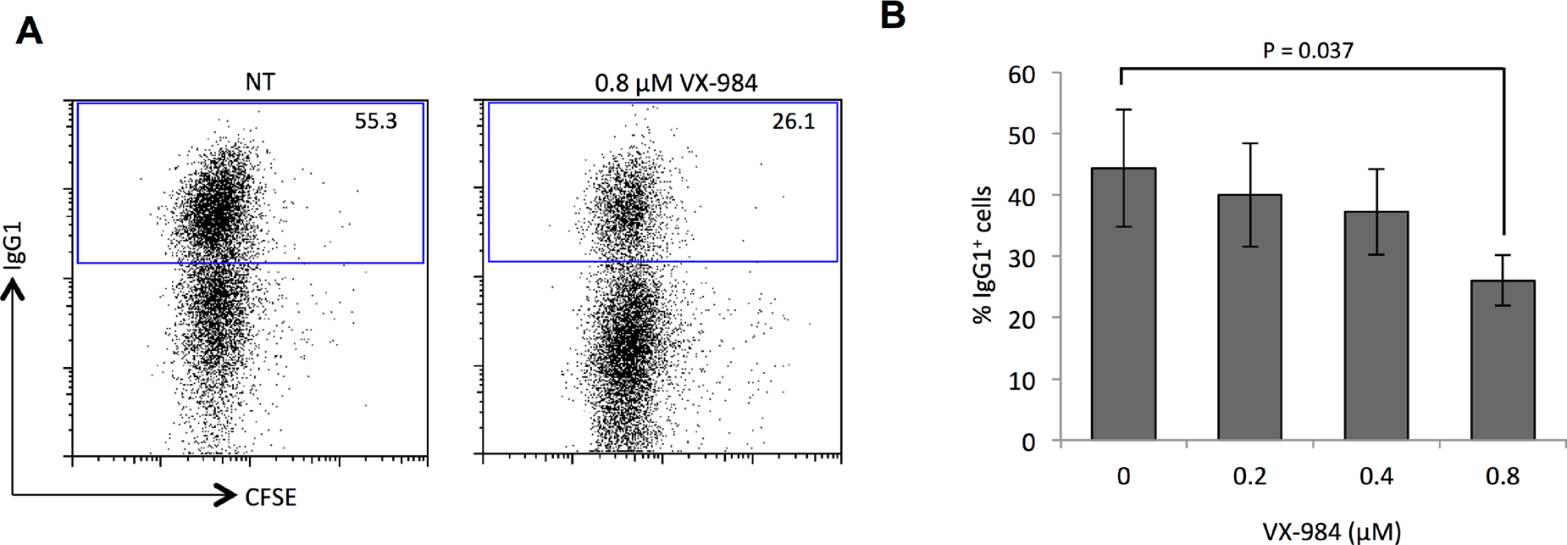
VX-984 reduces efficiency of NHEJ-dependent class switch recombination **(A)** Flow cytometry analysis of murine B cells cultured either with no treatment (NT) or in the presence of VX-984. Gated population indicates IgG1^+^ cells as percentage of total live population after three days of activation *in vitro*. CFSE fluorescence reveals cell proliferation was equivalent in the untreated and VX-984-treated cells. **(B)** Bar graph quantifying dose dependent reduction in class switch recombination efficiency in the presence of VX-984. Error bars show the standard deviation of the mean of N=3 experiments. P value was calculated using unpaired Student t test.

B cells in mammalian species use class switch recombination (CSR) to diversify the isotypes of immunoglobulin that are produced upon antigen-mediated activation. CSR involves induction of DSBs at immunoglobulin loci, followed by rejoining of DNA by NHEJ pathways. DNA-PKcs is essential for normal CSR [16], and the rate of CSR drops to around half of the normal level in cells lacking functional DNA-PKcs [17–19] We hypothesized that VX-984-mediated inhibition of DNA-PKcs could have the same effect as loss of the protein. We therefore measured CSR in primary murine B lymphocytes cultured *in vitro* in the presence of varying doses of VX-984 (Figure 1A-1B). We found a dose-dependent reduction in CSR efficiency with increasing VX-984 concentration. This result suggests that VX-984 can strongly block physiological NHEJ-dependent processes, presumably by inhibition of DNA-PKcs.

### VX-984 increases alternate pathways of DSB repair, including HR and mutagenic NHEJ (mNHEJ)

**Figure 2:**
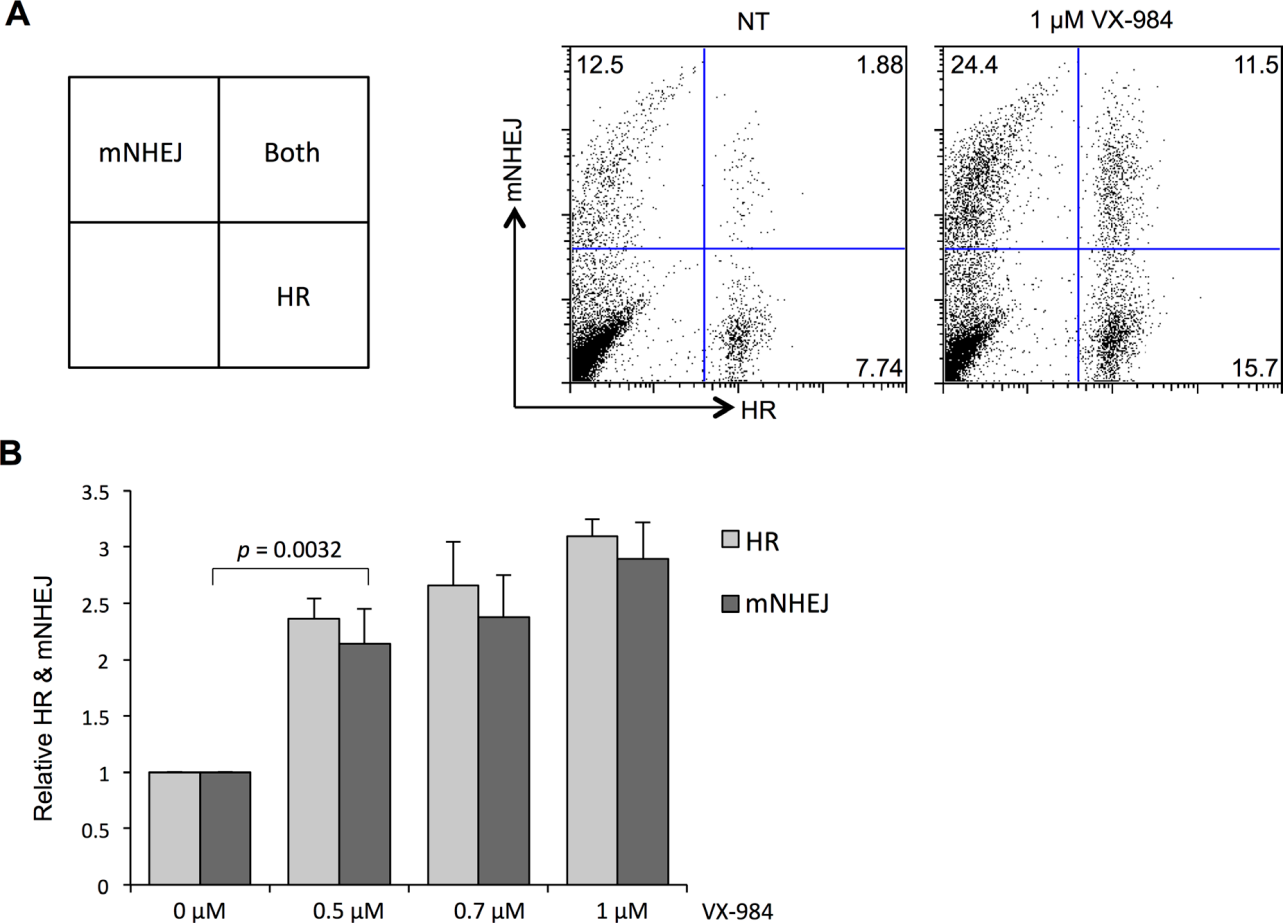
Altered use of DSB repair pathways in EJ-DR cells following VX-984 treatment **(A)** Flow cytometry analysis of EJ-DR reporter cell line following chemically-induced, I-SceI-mediated DSBs. GFP vs DsRed fluorescence reveals relative use of mutagenic NHEJ (mNHEJ) and HR as shown in the scheme. Repair was measured in cells that were not treated (NT) or treated with VX-984. **(B)** Quantification of flow cytometry results. Rates of HR and NHEJ are revealed to be significantly higher in cells treated with the putative DNA-PKcs inhibitor, VX-984.

NHEJ can proceed via a ‘classical’ pathway, involving DNA-PK, or by ‘alternative’ pathways, which have greater mutagenic potential [20]. We hypothesized that inhibition of DNA-PK-dependent classical NHEJ would result in compensatory utilization of alternate pathways for DSB repair. To test this hypothesis, we used the U2OS EJ-DR reporter cell line, which contains stably-integrated reporter constructs to measure the cellular response to DSBs. HR activity is revealed by GFP fluorescence in these cells, whereas mutagenic NHEJ is revealed by induced DsRed fluorescence [9]. We found significant dose-dependent increases in HR and mutagenic NHEJ when cells were pre-treated with VX-984 (Figure 2A-2B). This result is consistent with the idea that cells increase their use of alternative, potentially-mutagenic pathways for DSB repair following inhibition of the classical NHEJ pathway.

### VX-984 preferentially impairs resolution of DSBs in malignant astrocytes compared to normal astrocytes

**Figure 3:**
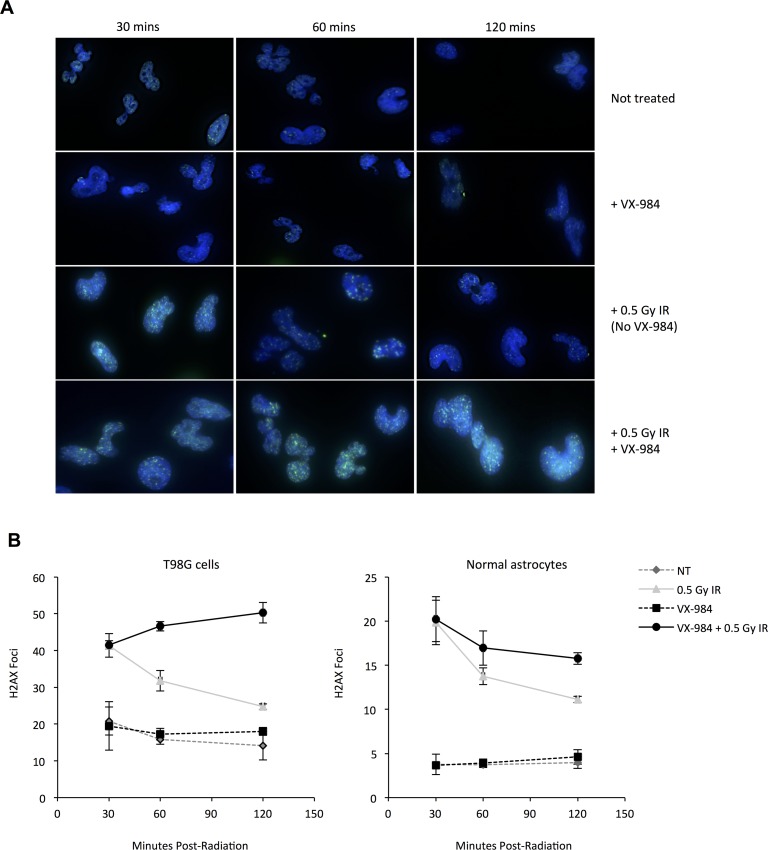
Altered resolution of γ-H2AX foci in cells following VX-984 treatment **(A)** Representative immunofluorescence images from normal and malignant cells treated with IR alone or with IR + VX-984. Nuclear foci of γ-H2AX (green spots) were quantified as a measure of unresolved DSBs. Blue staining indicates DAPI counter-stain of nuclear DNA. Labels indicate time post-IR treatment. γ-H2AX foci were induced 30 mins post-IR, and subsequently resolved as repair proceeded. **(B)** Bar graph representation of IR-induced γ-H2AX foci resolution over time with and without drug in transformed T98G glioma cells and normal human astrocytes.

One concern with an NHEJ inhibitor combined with a source for DSB (i.e. IR) is the possibility of normal tissue toxicities. We compared the dynamics of DSB repair after IR using foci of γ-H2AX as a read-out (Figure 3A). γ-H2AX is a phosphorylated form of the variant histone H2AX, and is induced in chromatin adjacent to DSBs, and subsequently resolved after DNA repair [21]. VX-984 alone did not induce γ-H2AX foci. The initial number of foci (at 30 minutes) were similar with 0.5 Gy alone or with VX-984 in both T98G (41.51 vs. 41.38; p>0.9999) and normal astrocytes (20.22 vs. 19.85; p = 0.9859) suggesting the drug did not increase the absolute number of DSBs. With radiation alone, DSB repair from 30 to 60 minutes (41.38 vs. 31.79; p = 0.0016) and from 60 to 120 minutes (31.79 vs. 24.75; p = 0.0167) was rapid and significant in T98G cells. In contrast, cells treated with VX-984 failed to resolve DSBs from 30 to 60 minutes (41.51 vs. 46.6; p = 0.09) and from 60 to 120 minutes (46.6 vs. 50.31; p = 0.2544). In irradiated normal astrocytes, there was similarly rapid DSB repair from 30 to 60 minutes (19.85 vs. 13.76; p < 0.0001) and from 60 to 120 minutes (13.76 vs. 11.12; p = 0.0093). However, normal astrocytes irradiated in the presence of VX-984 showed significant DSB repair from 30 to 60 minutes (20.22 vs. 16.95; p = 0.0017) and additional non-statistically significant repair from 60 to 120 minutes (16.95 vs 15.77; p=0.3042). This result suggests that VX-984 could be used to preferentially sensitize malignant cells to the clastogenic effects of radiotherapy.

### Inhibition of phosphorylation of DNA-PKcs substrates in a cancer cell line

**Figure 4:**
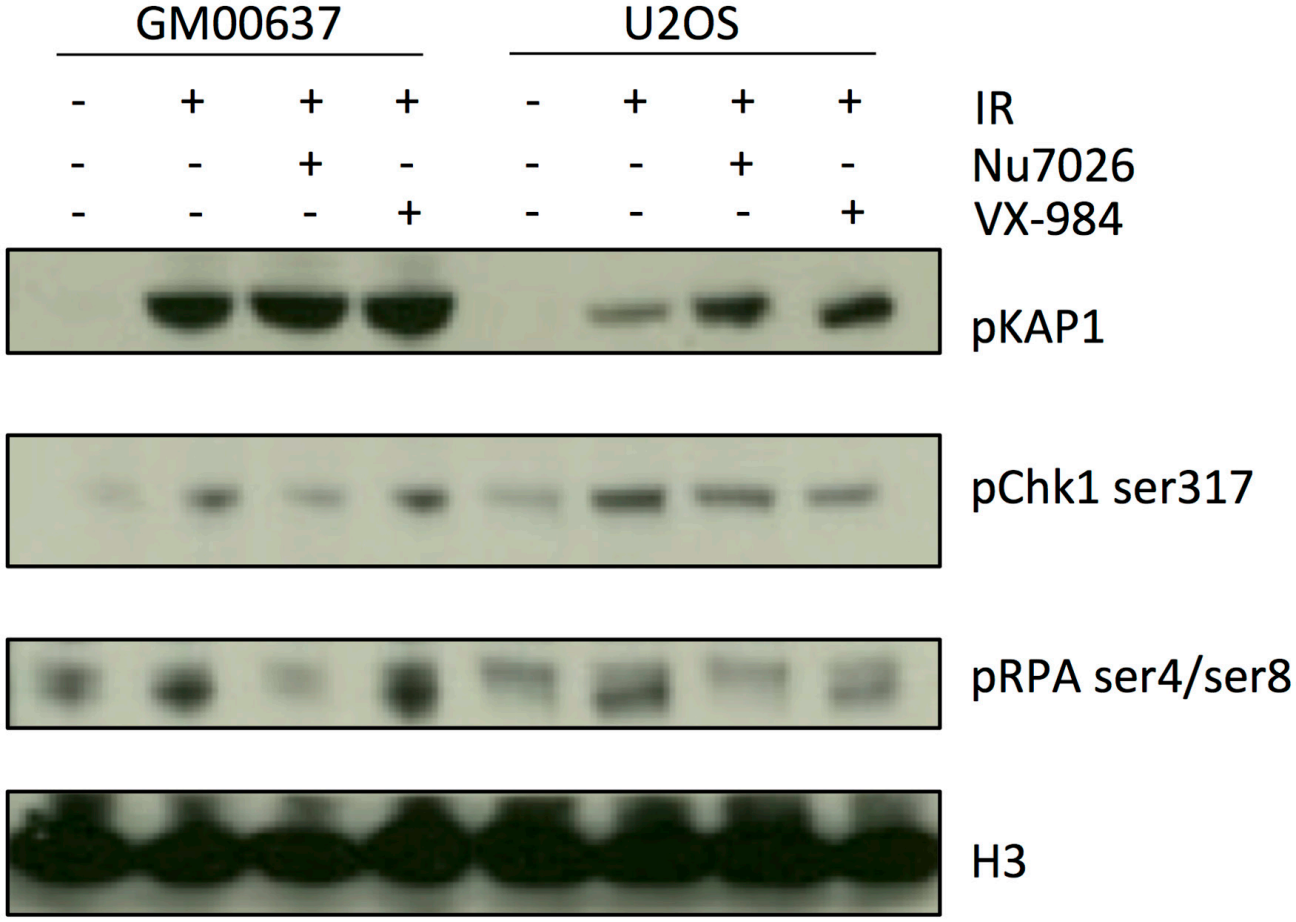
VX-984 inhibits phosphorylation of the DNA-PKcs substrate, RPA-Ser4/Ser8 in transformed cells Western blotting revealed the extent of phosphorylation of cellular components following IR treatment in cells that were not treated, or treated with the DNA-PKcs inhibitors, Nu7026 or VX-984.

To test the effects of VX-984 treatment on DSB signaling pathways, we performed Western blotting with protein lysates from cells treated with ionizing radiation and either VX-984 or the previously-characterized DNA-PKcs inhibitor, NU7026 [22]. For this experiment we used normal, untransformed human fibroblasts (GM00637, ATCC) or the U2OS osteosarcoma cell line. Following IR treatment, phosphorylation of KAP1, Chk1 Ser-317 and RPA-Ser4/Ser8 was observed, consistent with activation of ATM, ATR and DNA-PKcs respectively (Figure 4). As expected, IR-induced phosphorylation of the DNA-PKcs substrate RPA-Ser4/Ser8 was blocked by pretreatment with Nu7026. VX-984 treatment also reduced the degree of RPA-Ser4/Ser8 phosphorylation, but this effect appeared to be limited to the U2OS osteosarcoma cells, and was not evident in the untransformed GM00637 cells.

## DISCUSSION

DNA-PK is a PI3K-like kinase (PI3KK) that is comprised of the Ku70/Ku80 heterodimer and the DNA-PK catalytic subunit (DNA-PKcs). It shares significant homology with other PI3K family members including its more closely related serine/threonine kinases Ataxia-telangiectasia mutated (ATM) and ATM-and *Rad3-related* (ATR) [23]. DSB break repair by cNHEJ is initiated when the ring-like Ku70/80 heterodimers bind to each of the two broken ends of DNA. A pair of DNA-PKcs units then localize to the DSB through interactions with both the KU complex and with DNA, thus tethering the DSB. The assembled DNA-PK now amplifies the NHEJ signal through a series of auto- and exo- phosphorylation events. There are several dozen known autophosphorylation sites that include the well-described ABCDE cluster (2609-2647) and the PQR cluster (2023-2056) [24, 25]. Several elegant mutation studies have characterized the function and interdependence of these two autophosporylation targets [25–30]. DNA-PK is intimately involved and appears to orchestrate critical steps in NHEJ from end processing (through phosphorylation of WRN [31] and Artemis [32]) to the ligation steps mediated by DNA ligase IV/XRCC4 [33]. Still, the activity of DNA-PK appears to extend well beyond NHEJ; DNA-PK appears to cap and protect telomeric DNA and regulates p53-mediated apoptosis in certain contexts [4]. Therefore, a pharmacologic inhibitor or modulator of DNA-PK could be of significant value in the treatment of malignancies.

VX-984 is a selective and potent inhibitor of DNA-PKcs, and its effects were recently demonstrated in non-small cell lung cancer cell lines and primary samples by Boucher and colleagues [15]. In their report, VX-984 showed high selectivity for DNA-PKcs over other PI3Ks, and there was significant radiosensitization in the NSCLC cells tested *in vitro* and *in vivo* assays. Intriguingly, 6 of 7 normal cell lines (fibroblasts and epithelial cells) exhibited no radiosensitization, and xenograft-bearing mice demonstrated no loss of body weight and no signs of distress. While rapidly proliferating cells with multiple replication forks and high mitotic rates may be preferentially sensitive to HR inhibitors, NHEJ inhibitors could be expected to affect cycling and non-cycling cells similarly, given the activity of NHEJ in genome maintenance throughout the cell cycle. Thus the report from Boucher et al is interesting in the specificity of VX-984 for cancer cells.

In the current report we confirmed NHEJ inhibition with VX-984 and found that DSB repair appeared to be impaired preferentially in T98G cells compared to the normal astrocyte cells. Thus our report is in line with the findings of Boucher et al [15] and taken together, our findings appear to support some therapeutic specificity of VX-984 for transformed cells; however the precise mechanism for this will need further study. Based on our analysis of DSB signaling pathways, it appears that DNA-PKcs signaling can be blocked more easily in transformed cells than in normal cells, as revealed by diminished phosphorylation of RPA-Ser4/Ser8 in these cells following IR treatment in the presence of VX-984. It is possible that in these cancer cells, there is altered expression or activity of DSB signaling molecules such as ATM or ATR, which makes these cells particularly sensitive to VX-984-mediated DNA-PKcs inhibition. This effect may account for the increased cytotoxic effect of VX-984 in transformed cells. We demonstrated also that inhibition of NHEJ by VX-984 activated alternate repair pathways including HR; this raises the tantalizing prospect that some cells may engage this switch more proficiently than others, making for a plausible and testable hypothesis for therapeutic specificity.

There are important limitations to our work. First, this report does not definitively demonstrate a difference in activity between transformed and untransformed cells. It does incrementally advance the findings of Boucher et al [15] in this regard. Second, this work will need to be expanded into multiple cells lines and will have to withstand the rigor of repetitive testing in different labs and across disease types. Cell lethality experiments (such as clonogenic assays) in isogenic pairs will need to corroborate the findings from this report.

There has recently been great interest in immune checkpoint inhibitors that disinhibit host T-cells to produce anti-tumor effects. Rizvi and colleagues performed whole exome sequencing on a cohort of non-small cell lung cancer patients treated with pembrolizumab, an antibody targeting PD-1 (programmed cell death-1) [34]. They found that patients with higher loads of non-synonymous mutations were more likely to have durable response to pembrolizumab in both discovery and validation cohorts. Interestingly, they found that tumors harboring the greatest mutation burdens often had deleterious mutations in genes involved in DNA repair such as *POLD1, POLE*, and *PRKDC* (the gene encoding DNAPKcs). This leads one to the provocative and testable hypothesis that drugs that impair DDR, such as VX-984, may increase the mutational load and the resultant neoantigens that predict for response to checkpoint inhibitors. If true, patients who are non-responders to immunotherapy could be treated with these drugs for their direct therapeutic effect but also with a plan to re-challenge with immunotherapy later. Additional work will be required to study this potential effect and the time dynamics.

## MATERIALS AND METHODS

### Drug

VX-984 was obtained under an MTA for research purposes from Vertex Pharmaceuticals, Boston MA.

### Commercial cell lines

The T98G glioblastoma multiforme cell line (ATCC CRL-1690) was obtained from ATCC and cultured based on the specifications provided by ATCC. The human astrocyte cell line (ScienCell 1800) was purchased from ScienCell and was similarly cultured based upon the directions provided by ScienCell. GM00637 normal human fibroblasts, SV-40 transformed (Coriell) and U2OS human bone osteosarcoma epithelial cells (ATCC HTB-96) were cultured in DMEM (Sigma D5796) containing 10% FBS (Gemini 100-106) and 1% penicillin/streptomycin (Gibco 15070063).

### Culture of human astrocytes

T98G cell cultures were grown and subsequently passaged in 75 cm^2^ tissue culture flasks from Azer Scientific. T98G was incubated at 37°C in the presence of EMEM with L-glutamine containing 10% fetal bovine serum and penicillin/streptomycin from ATCC. Dulbecco's Phosphate Buffered Saline (DPBS) from Sigma Aldrich and 0.25% Trypsin-EDTA from Gibco were used in the passage of T98G. T98G was passaged three times after initial thawing prior to use in any experiments.

The human astrocytes were also cultured in 75 cm^2^ tissue culture flasks from Azer Scientific. The flasks were coated with 15 μL of poly-L-lysine and incubated in 37°C for a minimum of one hour to improve the adhesion of cells. The astrocytes were cultured in astrocyte media, which contained fetal bovine serum, penicillin/streptomycin, and Astrocyte Growth Supplement. The astrocytes were passaged according to the protocol provided by the vendor.

### Class switch recombination assay

B cells were isolated by lysing red blood cells with ACK lysing buffer, followed by reverse selection using anti-CD43 MACS micro-beads (Miltenyi Biotech 130-049-801) and a Mini-MACS column. Collected B cells were stained with 5 μM CFSE for 10 minutes at 37°C and then cultured in RPMI-1640 containing 10% FBS, 1x penicillin/streptomycin, 1% L-glutamine, 1x MEM non-essential amino acids, 1% sodium pyruvate, 1% HEPES, and 53 mM 2-mercaptoethanol. Media was supplemented with 25 μg/ml LPS from E.coli (Sigma L2630), 50 U/ml Interleukin-4 (Sigma I1020), and 1:1000 rat anti-mouse anti-CD180 (BD Pharmingen 552128). Cells were cultured for 72 hours with VX-984 (0.2, 0.4, and 0.8 μM) at 37°C with 5% CO_2_.

B cells were blocked with 5 μl of anti-mouse CD16/CD32 (BD pharmingen 553141). Primary antibodies Biotin-rat anti-mouse IgG1 (BD Pharmingen 550331) and FITC-rat anti-mouse CD45R/B220 (BD Pharmingen 553088) were added at 10 μl of a 1:100 dilution, followed by 10 μl of a 1:100 dilution of Streptavidin-A647 (Life Technologies S32357). Cell proliferation and IgG+ cells were measured using a Becton Dickinson FACSCalibur and data were analyzed by FlowJo software.

### End-joining dual reporter (EJ-DR) assay

U2OS EJ-DR cells [9] (A gift from Dr. Shridar Ganesan, clone 34) were plated in a 6 well plate in DMEM containing 10% charcoal stripped FBS and 1% antibiotic-antimycotic. After 72 hours, the cells were washed and the medium was replaced with DMEM containing 10% tet-free FBS and 1% antibiotic-antimycotic and VX-984 (0.5, 0.7, and 1.0 μM) was added. To induce double-strand breaks at the I-Sce1 sites, 1 μM Shield1 (Clontech 632189) and 100 nM Triamcinolone Acetonide were added 30 minutes after drug addition. After 24 hours, cells were washed and fresh EJ-DR Tet-free medium and drug was added. After 72 hours, cells were collected and the levels of HR and NHEJ were measured using a Becton Dickinson FACSCalibur and data were analyzed by FlowJo software.

### Immunofluorescence assay

This assay was used to visualize the presence and ensuing resolution of H2AX foci following DNA damage with and without VX-984 over time. The level of phosphorylated histone variant H2AX (γ-H2AX) is a surrogate marker for levels of DNA damage. Immediately after double strand breakage (DSB), γH2AX forms bright nuclear foci that can be stained and visualized by immunoflourescent microscopy. The presence of γ-H2AX foci is widely regarded as marker for DSBs in cells [35–37] and has been shown to be specific for DSB [38]. We used the antibody to γ-H2AX (Millipore) to visualize cells with DNA damage.

T98G and human astrocyte cells were plated onto BD Falcon Culture Slides treated with either 0.5 Gy irradiation treatment or no irradiation. Slides containing the human astrocytes were coated with 1μL of poly-Lysine for one hour. After incubation overnight, 1μM VX-984 was given to cells in wells designated for treatment, while the control wells were given fresh media. After one hour of incubation with VX-984, the slides were irradiated at 0.5 Gy using our irradiator. Immunofluorescence staining occurred thirty minutes, one hour, and two hours following radiation. The cells were fixed with buffered formaldehyde (SF 93-4) from Fisher Scientific for fifteen minutes and then permeabilized with 0.02% Triton for ten minutes. The cells were then blocked in a 3% albumin in DPBS (Sigma) for one hour. Primary antibody against γ-H2AX was added in a 1:400 dilution in the 3% albumin solution overnight and the slides were incubated at 4°C. Fluorescent anti-rabbit IgG secondary antibody (Vector) was then added in a 1:300 concentration in albumin. The slides were kept in darkness for one hour prior to washing. The slides were mounted using Vectashield Mounting Medium for Fluorescence with DAPI, (VectorShield), prior to imaging under a fluorescent microscope. Once the slides were imaged, 100 cells per condition were counted to obtain γH2AX foci numbers. The assay was repeated in triplicate.

### Western blotting

For western blotting, primary antibodies were used at the following dilutions: anti-pKAP1 (1:800, Bethyl A300-767A), anti-pChk1 ser317 (1:500, Cell Signaling 2344S), anti-pRPA32 ser4/ser8 (1:500, Bethyl A300-245A), anti-histone H3 (1:10,000, AbCam 1791).

### Statistics

GraphPad Prism v.7.01 software was used for statistical analysis. IF assays were assessed using 2-way ANOVA. Between group's differences were confirmed using Turkey's multiple comparisons test. A p-value of ≤ 0.05 was considered significant. For Class Switch Recombination assay, statistical significance was confirmed using unpaired Student t test.

## Abbreviations

BSA: bovine serum albumin
DSB: double-strand break
IR: Ionizing radiation
HR: homologous recombination
DNA-PK: DNA-dependent protein kinase
DNA-PKcs: catalytic subunit of
DNA: dependent protein kinase
NHEJ: non-homologous end joining
RT: radiation therapy.

## References

[1] Thompson LH (2012). Recognition, signaling, and repair of DNA double-strand breaks produced by ionizing radiation in mammalian cells: the molecular choreography. Mutat Res.

[2] Jackson SP, Bartek J (2009). The DNA-damage response in human biology and disease. Nature.

[3] Helleday T, Petermann E, Lundin C, Hodgson B, Sharma RA (2008). DNA repair pathways as targets for cancer therapy. Nat Rev Cancer.

[4] Hill R, Lee PW (2010). The DNA-dependent protein kinase (DNA-PK): more than just a case of making ends meet?. Cell Cycle.

[5] Davidson D, Amrein L, Panasci L, Aloyz R (2013). Small Molecules, Inhibitors of DNA-PK, Targeting DNA Repair, and Beyond. Front Pharmacol.

[6] Dobbs TA, Tainer JA, Lees-Miller SP (2010). A structural model for regulation of NHEJ by DNA-PKcs autophosphorylation. DNA Repair (Amst).

[7] Johnson RD, Jasin M (2001). Double-strand-break-induced homologous recombination in mammalian cells. Biochem Soc Trans.

[8] Radhakrishnan SK, Jette N, Lees-Miller SP (2014). Non-homologous end joining: emerging themes and unanswered questions. DNA Repair (Amst).

[9] Bindra RS, Goglia AG, Jasin M, Powell SN (2013). Development of an assay to measure mutagenic non-homologous end-joining repair activity in mammalian cells. Nucleic Acids Res.

[10] Goodwin JF, Knudsen KE (2014). Beyond DNA repair: DNA-PK function in cancer. Cancer Discov.

[11] Hammel M, Yu Y, Mahaney BL, Cai B, Ye R, Phipps BM, Rambo RP, Hura GL, Pelikan M, So S, Abolfath RM, Chen DJ, Lees-Miller SP, Tainer JA (2010). Ku and DNA-dependent protein kinase dynamic conformations and assembly regulate DNA binding and the initial non-homologous end joining complex. J Biol Chem.

[12] Davis AJ, So S, Chen DJ (2010). Dynamics of the PI3K-like protein kinase members ATM and DNA-PKcs at DNA double strand breaks. Cell Cycle.

[13] Azad A, Jackson S, Cullinane C, Natoli A, Neilsen PM, Callen DF, Maira SM, Hackl W, McArthur GA, Solomon B (2011). Inhibition of DNA-dependent protein kinase induces accelerated senescence in irradiated human cancer cells. Mol Cancer Res.

[14] Davidson D, Grenier J, Martinez-Marignac V, Amrein L, Shawi M, Tokars M, Aloyz R, Panasci L (2012). Effects of the novel DNA dependent protein kinase inhibitor, IC486241, on the DNA damage response to doxorubicin and cisplatin in breast cancer cells. Invest New Drugs.

[15] Boucher D, Hoover R, Wang Y, Gu Y, Newsome D, Ford P, Moody C, Damagnez V, Arimoto R, Hillier S, Wood M, Markland W, Eustace B Potent radiation enhancement with VX-984, a selective DNA-PKcs inhibitor for the treatment of NSCLC.

[16] Jolly CJ, Cook AJ, Manis JP (2008). Fixing DNA breaks during class switch recombination. J Exp Med.

[17] Manis JP, Dudley D, Kaylor L, Alt FW (2002). IgH class switch recombination to IgG1 in DNA-PKcs-deficient B cells. Immunity.

[18] Franco S, Murphy MM, Li G, Borjeson T, Boboila C, Alt FW (2008). DNA-PKcs and Artemis function in the end-joining phase of immunoglobulin heavy chain class switch recombination. J Exp Med.

[19] Cook AJ, Oganesian L, Harumal P, Basten A, Brink R, Jolly CJ (2003). Reduced switching in SCID B cells is associated with altered somatic mutation of recombined S regions. J Immunol.

[20] Bunting SF, Nussenzweig A (2013). End-joining, translocations and cancer. Nat Rev Cancer.

[21] Rogakou EP, Pilch DR, Orr AH, Ivanova VS, Bonner WM (1998). DNA double-stranded breaks induce histone H2AX phosphorylation on serine 139. J Biol Chem.

[22] Willmore E, de Caux S, Sunter NJ, Tilby MJ, Jackson GH, Austin CA, Durkacz BW (2004). A novel DNA-dependent protein kinase inhibitor, NU7026, potentiates the cytotoxicity of topoisomerase II poisons used in the treatment of leukemia. Blood.

[23] Smith GC, Divecha N, Lakin ND, Jackson SP (1999). DNA-dependent protein kinase and related proteins. Biochem Soc Symp.

[24] Douglas P, Cui X, Block WD, Yu Y, Gupta S, Ding Q, Ye R, Morrice N, Lees-Miller SP, Meek K (2007). The DNA-dependent protein kinase catalytic subunit is phosphorylated in vivo on threonine 3950, a highly conserved amino acid in the protein kinase domain. Mol Cell Biol.

[25] Meek K, Douglas P, Cui X, Ding Q, Lees-Miller SP (2007). Autophosphorylation at DNA-dependent protein kinase's two major autophosphorylation site clusters facilitates end processing but not end joining. Mol Cell Biol.

[26] Block WD, Yu Y, Merkle D, Gifford JL, Ding Q, Meek K, Lees-Miller SP (2004). Autophosphorylation-dependent remodeling of the DNA-dependent protein kinase catalytic subunit regulates ligation of DNA ends. Nucleic Acids Res.

[27] Chen BP, Chan DW, Kobayashi J, Burma S, Asaithamby A, Morotomi-Yano K, Botvinick E, Qin J, Chen DJ (2005). Cell cycle dependence of DNA-dependent protein kinase phosphorylation in response to DNA double strand breaks. J Biol Chem.

[28] Cui X, Yu Y, Gupta S, Cho YM, Lees-Miller SP, Meek K (2005). Autophosphorylation of DNA-dependent protein kinase regulates DNA end processing and may also alter double-strand break repair pathway choice. Mol Cell Biol.

[29] Ding Q, Reddy YV, Wang W, Woods T, Douglas P, Ramsden DA, Lees-Miller SP, Meek K (2003). Autophosphorylation of the catalytic subunit of the DNA-dependent protein kinase is required for efficient end processing during DNA double-strand break repair. Mol Cell Biol.

[30] Reddy YV, Ding Q, Lees-Miller SP, Meek K, Ramsden DA (2004). Non-homologous end joining requires that the DNA-PK complex undergo an autophosphorylation-dependent rearrangement at DNA ends. J Biol Chem.

[31] Yannone SM, Roy S, Chan DW, Murphy MB, Huang S, Campisi J, Chen DJ (2001). Werner syndrome protein is regulated and phosphorylated by DNA-dependent protein kinase. J Biol Chem.

[32] Yannone SM, Khan IS, Zhou RZ, Zhou T, Valerie K, Povirk LF (2008). Coordinate 5′ and 3′ endonucleolytic trimming of terminally blocked blunt DNA double-strand break ends by Artemis nuclease and DNA-dependent protein kinase. Nucleic Acids Res.

[33] Hsu HL, Yannone SM, Chen DJ (2002). Defining interactions between DNA-PK and ligase IV/XRCC4. DNA Repair (Amst).

[34] Rizvi NA, Hellmann MD, Snyder A, Kvistborg P, Makarov V, Havel JJ, Lee W, Yuan J, Wong P, Ho TS, Miller ML, Rekhtman N, Moreira AL (2015). Cancer immunology. Mutational landscape determines sensitivity to PD-1 blockade in non-small cell lung cancer. Science.

[35] Fillingham J, Keogh M, Krogan NJ (2006). Gamma H2AX and its role in DNA double-strand break repair. Biochem Cell Biol.

[36] Pilch DR, Sedelnikova OA, Redon C, Celeste A, Nussenzweig A, Bonner WM (2003). Characteristics of gamma-H2AX foci at DNA double-strand breaks sites. Biochem Cell Biol.

[37] Sedelnikova OA, Rogakou EP, Panyutin IG, Bonner WM (2002). Quantitative detection of (125)IdU-induced DNA double-strand breaks with gamma-H2AX antibody. Radiat Res.

[38] Takahashi A, Ohnishi T (2005). Does gammaH2AX foci formation depend on the presence of DNA double strand breaks?. Cancer Lett.

